# Sub-toxic events induced by truck speed-facilitated PM_2.5_ and its counteraction by epigallocatechin-3-gallate in A549 human lung cells

**DOI:** 10.1038/s41598-022-18918-x

**Published:** 2022-09-02

**Authors:** Shih Yu Pan, Kai Hsien Chi, Yen-Cih Wang, Wen-Chi Wei, Yune-Fang Ueng

**Affiliations:** 1 Institute of Environmental and Occupational Health Sciences, National Yang Ming Chiao Tung University, Taipei, 112 Taiwan, ROC; 2 Division of Basic Chinese Medicine, National Research Institute of Chinese Medicine, 155-1, Li-Nong Street, Sec. 2, Taipei, 112 Taiwan, ROC; 3 Graduate Institute of Medical Sciences, College of Medicine, Taipei Medical University, Taipei, 110 Taiwan, ROC; 4 Cell Physiology and Molecular Image Research Center, Wan Fang Hospital, Taipei Medical University, Taipei, Taiwan, ROC; 5 Institute of Biopharmaceutical Science, School of Pharmacy, National Yang Ming Chiao Tung University, Taipei, 112 Taiwan, ROC

**Keywords:** Chemical biology, Environmental sciences, Biomarkers, Risk factors

## Abstract

To distinguish the influences of fuel type and truck speed on chemical composition and sub-toxic effects of particulates (PM_2.5_) from engine emissions, biomarkers—interleukin-6 (IL-6), cytochrome P450 (CYP) 1A1, heme oxygenase (HO)-1, and NADPH-quinone oxidoreductase (NQO)-1—were studied in A549 human lung cells. Fuel type and truck speed preferentially affected the quantity and ion/polycyclic aromatic hydrocarbon (PAH) composition of PM_2.5_, respectively. Under idling operation, phenanthrene was the most abundant PAH. At high speed, more than 50% of the PAHs had high molecular weight (HMW), of which benzo[*a*]pyrene (B[a]P), benzo[*ghi*]perylene (B[ghi]P), and indeno[1,2,3-*cd*]pyrene (I[cd]P) were the main PAHs. B[a]P, B[ghi]P, and I[cd]P caused potent induction of IL-6, CYP1A1, and NQO-1, whereas phenanthrene mildly induced CYP1A1. Based on the PAH-mediated induction, the predicted increases in biomarkers were positively correlated with the measured increases. HMW-PAHs contribute to the biomarker induction by PM_2.5_, at high speed, which was reduced by co-exposure to epigallocatechin-3-gallate.

## Introduction

Inhalation of small airborne particulate matter with an aerodynamic diameter smaller than 2.5 μm (PM_2.5_) is linked to various diseases including nasal inflammation, pollen-induced allergic rhinitis, and cancer^1,2^. PM_2.5_ comprises a main mixture of inorganic, carbonaceous, and metallic substances^1^, as well as biological substances, such as pollens and microorganisms, which can be absorbed on the surface^3,4^. PM_2.5_ with the small size can deposit in lower respiratory tissues including bronchi and affect the gas exchange within lungs, leading to a significant health problem. Heavy metals, polycyclic aromatic hydrocarbons (PAHs) and endotoxins make the main contribution to the PM-induced toxicities in respiratory, nervous and cardiovascular systems^5^. The main factors of PM_2.5_-elicited inflammation and carcinogenesis include oxidative stress, the activation of the aryl hydrocarbon receptor (AhR), and cytokine release, which are all defensive responses^1,6^. However, sustained AhR activation/oxidative stress and cytokine stimulation may cause airway injury, such as chronic bronchitis^7^. For example, the potent AhR activation by 2,3,7,8-tetrachlorodibenzo-*p*-doxin increased pulmonary neutrophil influx and mortality in mice with influenza virus infection^8,9^. PAHs differentially induce cytochrome P450 (CYP) 1A1, which is present at a high level in the respiratory tract and is responsible for both the detoxification and mutagenic activation of some PAHs, such as, B[a]P^10^. CYP1A1 showed high responsiveness to the AhR activation. PAHs, including benzo[*a*]pyrene (B[a]P), play important roles in the toxicity of PM_2.5_, with the inappropriate activation of AhR by PAHs contributing to particulate matter-linked cardiotoxicity^6^ and lung diseases^7^. Both heme oxygenase (HO)-1 and NADPH-quinone oxidoreductase (NQO)-1 are downstream targets of the AhR-cross-talked nuclear factor-erythroid 2-related factor 2 (Nrf2). HO-1 and NQO-1 are preferentially, but differentially, induced by oxidative stress-generating substances, such as heavy metals and cigarette smoke^11,12^. The induction of HO-1 and NQO-1 is crucial for fighting against the elevated reactive oxygen species (ROS), which are key factors in pulmonary inflammation^1^. The pro-inflammatory cytokine interleukin (IL)-6 is involved in the proliferation of fibroblasts and the advancement of pulmonary fibrosis^13^. As such, serum IL-6 levels positively correlate to poor clinical outcomes in patients with chronic obstructive pulmonary disease^14^. IL-6, CYP1A1, HO-1 and NQO-1 showed specific and cross-linked contribution to cellular protection/detrimental effects upon xenobiotic exposure. Therefore, the expression levels of each may be useful biomarkers for assessing exposure to airborne particulates of different compositions.

Vehicle exhaust has been identified as one of the main sources of PM_2.5_, as an aerosol, thereby presenting a global air pollution issue. In gasoline exhaust from cars, the order of PAH composition (w/w) in PM_2.5_ was low-molecular weight (LMW) (2 and 3 rings)-PAHs (67%) > medium-molecular-weight (MMW)-PAHs (4 rings) (19%) > high-molecular-weight (HMW)-PAHs (≥ 5 rings) (14%)^15^. A study on heavy trucks with diesel engines by the same group showed that the composition of LMW-PAHs was similar to the composition of HMW-PAHs at 34% and 32% respectively, with MMW-PAHs at 46%^16^. In a study of 5 light trucks, under idling operation, LMW-PAHs had the least mass abundance (~ 10%) in 4 trucks and one emitted similar abundance of LMW- and HMW-PAHs (~ 28%)^17^. Truck speed (50 km/h) has little influence on the abundance of HMW-PAHs in the total particles emitted. Another study revealed that the removal of fuel-after-treatment devices increased the abundance of particulate-bound HMW-PAHs in a heavy-duty truck^18^. When a catalyzed diesel particulate filter was introduced, LMW-PAHs (2 rings) were the most abundant particulate emitted from a truck under idling operation. However, the abundance of HMW-PAHs increased from < 1 to 7% as the truck speed increased to 80 km/h. As such, the influence of speed on the ionic and metallic composition of PM_2.5_ from vehicle emissions remains unclear. More evidence is required to reveal the association between the composition of PAHs in PM_2.5_, and the induction of the previously mentioned biomarkers.

Studies on epigallocatechin-3-gallate (EGCG) have revealed that this antioxidant found in green tea acts as an AhR antagonist via the reduction of AhR ligand-induced DNA binding^19,20^, and EGCG decreased the downstream transcriptional upregulation of CYP1A1 in hepatoma Hepa1c1c7 cells^17^. Both tea and EGCG ameliorated B[a]P-elevated serum biomarkers of hepatic damage, alanine and aspartate aminotransferases, in rats^21^. In addition, EGCG reduced PM_10_-elevated transcript levels of proinflammatory cytokines, tissue necrosis factor (TNF)-α, IL-6, IL-8, and IL-1β, in cryopreserved human epidermal keratinocytes (adult skin)^22^. The lung tissue may exhibit tissue-selective inflammatory responses and metabolic enzyme regulation^4,10,23,24^. As such, the potential protective effect of EGCG against inhaled PM_2.5_ requires further studies in lung cells.

**Figure 1:**
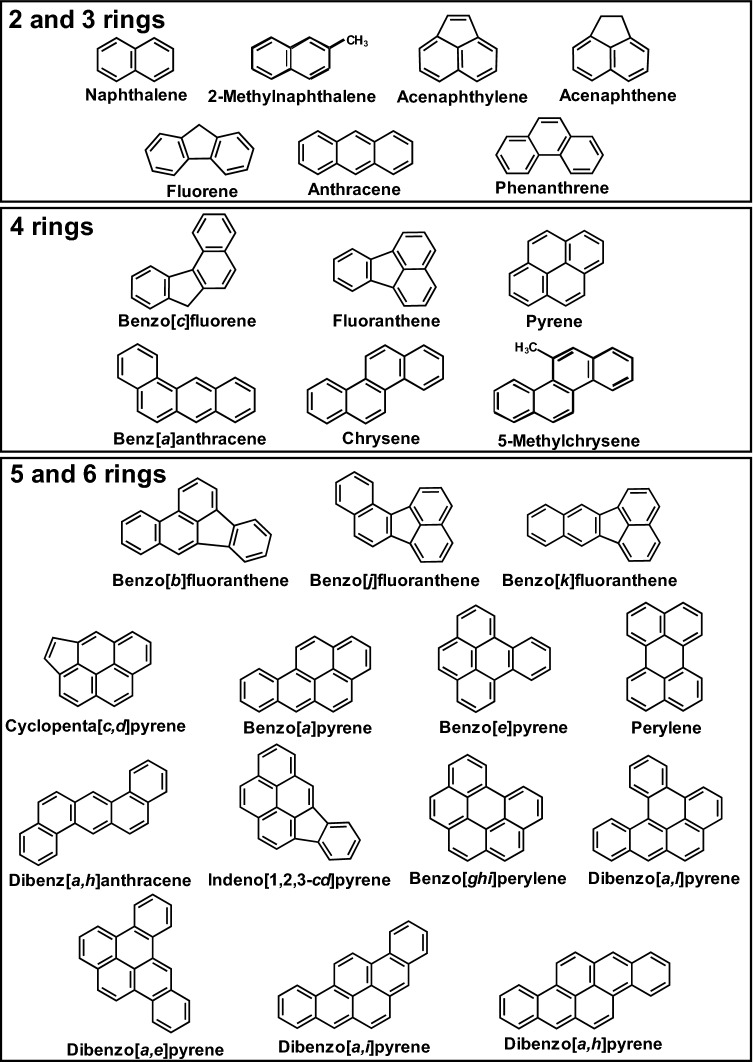
The chemical structures of the LMW (2 and 3 rings)-, MMW (4 rings)-, and HMW (5 and 6 rings)-PAHs.

Since humans are typically exposed to PM_2.5_ at sub-toxic levels, alterations in biomarkers IL-6, CYP1A1, HO-1, and NQO-1, may reflect the responses to sub-toxic PM_2.5_ exposures. Therefore, this study aimed to examine the influences of fuel types and mobile speeds on the exhaust composition-associated induction of biomarkers using human lung alveolar cell line A549. The ionic (10 ions), PAH (27 PAHs, Fig. 1), and metallic (46 metals) composition of PM_2.5_ in exhausts were quantitated. The main HMW-PAHs contributing to the speed relevant differential biomarker induction were identified. Additionally, the time-dependent effects of EGCG on the exposure markers induced by the PM_2.5_-bound PAHs, emitted from diesel engines at high speed (diesel-high), were evaluated.

## Results

### The differential emission contents and B[a]P toxicity equivalents of PM_2.5_ emitted from trucks at idle and high speeds

**Figure 2:**
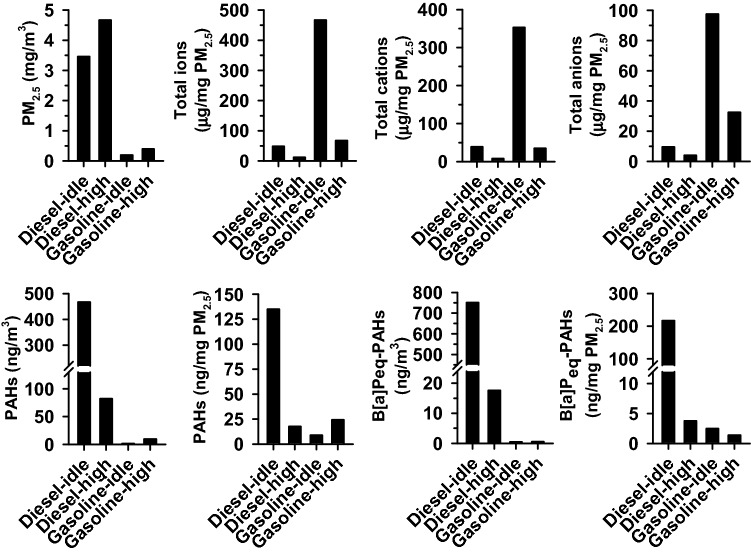
The influence of speed and diesel type on the composition and B[a]P toxicity equivalents (B[a]Peq) of PM _2.5_ in the exhausts. Exhausts were collected from trucks with gasoline or diesel engine under idle and high (70 km/h) speeds and the PM_2.5_ content and the total contents of ions and PAHs (∑_24-27_PAHs) in PM_2.5_ particulates were quantitated as described in the Methods.

Under idling operation, the diesel engine generated a PM_2.5_ level (mg/m^3^) 18-fold higher than that of the gasoline engine (Fig. 2). Similarly, at high-speed, diesel engines generated a PM_2.5_ level 12-fold higher than that of gasoline engines. Compared with the respective fuel emissions under idle condition, diesel-high and gasoline-high generated 35% and 108% higher concentration levels of PM_2.5_, respectively. The order of the total content of water-soluble ions (μg/mg PM_2.5_) was gasoline-idle > gasoline-high ≅ diesel-idle > diesel-high exhaust. Diesel-idle exhaust generated a 7.7-fold increase in the total PAH level (ng/mg PM_2.5_) compared with diesel-high exhaust, whereas gasoline-idle exhaust generated only 36% of the total PAH content compared with gasoline-high exhaust. Regarding PM_2.5_ composition, gasoline-idle exhaust contained the highest levels (μg/mg PM_2.5_) of ions, whereas the diesel-idle exhaust contained the highest levels of total PAHs. Consistent with the differences in the total PAH levels in PM_2.5_ of fuel emissions from trucks at idle and high speeds, the B[a]Peq level (ng/m^3^ or ng/mg PM_2.5_) of diesel-idle was greater than that of diesel-high, and the difference (> 40-fold) was greater than that between gasoline-idle and gasoline-high exhaust (1.2 ~ 1.8-fold).

### The differential ionic and PAH composition of PM_2.5_ in the fuel emissions

**Figure 3:**
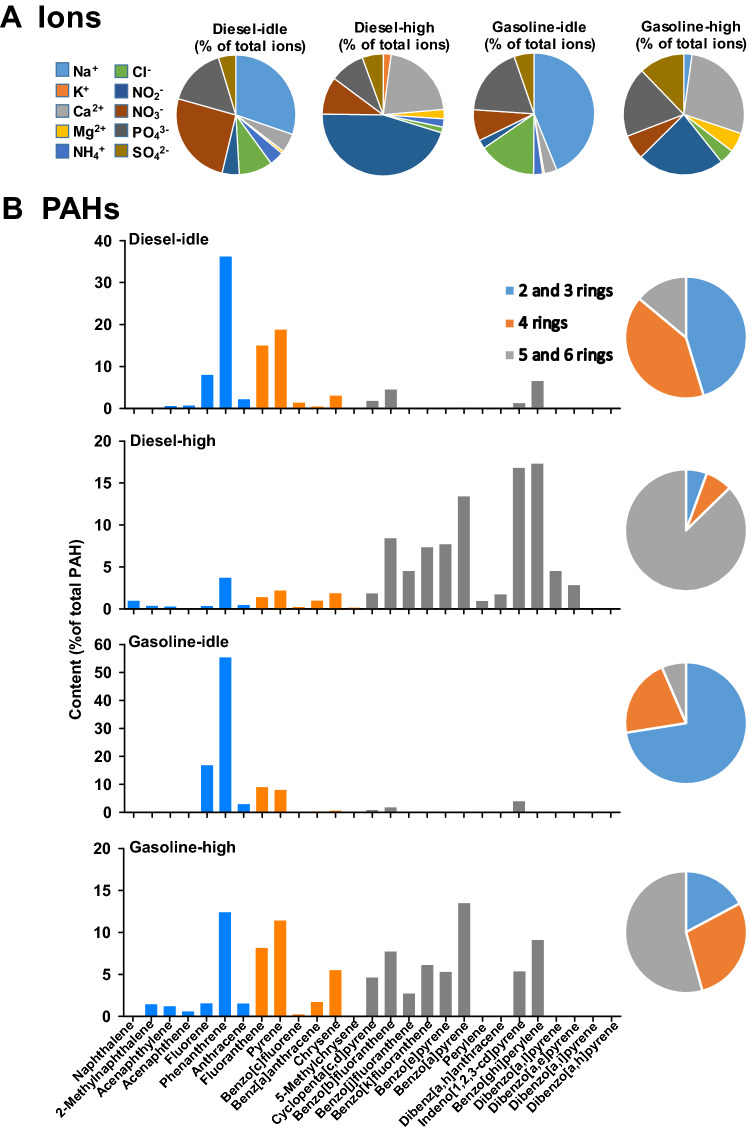
The relative abundance (%) of individual ion in total ions (**A**) and PAH in total PAHs (B) of PM_2.5_ particulates collected from trucks with gasoline or diesel engine under idle and high speeds. In panel (**B**), the percentages of the relative contents of the sub-groups of PAHs with low- (2 and 3 rings), medium- (4 rings), and high-molecular weight (5 and 6 rings) are shown in the pie chart on the right.

The results of the analysis of the ionic composition showed that the levels (μg/mg PM_2.5_) of individual ions in the gasoline exhaust were all higher than the respective levels in diesel exhausts under the same driving speed (Figs. 2 and 3A). When the truck engines ran under idling operation, Na^+^ was the most abundant cations in the fuel emissions (Fig. 3A). Among the cations, the order of relative ionic abundance was Na^+^  > Ca^2+^  > NH_4_^+^  > Mg^2+^  > K^+^ in both the diesel-idle and gasoline-idle exhausts. The order of relative abundance of anions was NO_3_^-^ > PO_4_^3−^ > Cl^−^ > SO_4_^2−^ ≈ NO_2_^−^ and PO_4_^3−^ > Cl^−^ > NO_3_^-^ > SO_4_^2−^ > NO_2_^−^ in the diesel-idle and gasoline-idle exhausts, respectively. Engine transition from idle to high speed decreased the contents of Na^+^ (diesel: 14.7 to 0.01 μg/mg PM_2.5_; gasoline: 205 to 1.45 μg/mg PM_2.5_), PO_4_^3−^ (diesel: 7.75 to 1.13 μg/mg PM_2.5_; gasoline: 86.8 to 12.3 μg/mg PM_2.5_) and NO_3_^-^ ions (diesel: 12.4 to 1.2 μg/mg PM_2.5_; gasoline: 38.9 to 4.4 μg/mg PM_2.5_), whereas K^+^ ion increased (diesel: a undetected level to 0.25 μg/mg PM_2.5_). An increase in the truck speed to 70 km/h, resulted in a change in the main cation (Na^+^ to Ca^2+^), and anion (PO_4_^3−^ or NO_3_^-^ to NO_2_^−^). The relative cationic abundance was in the order of Ca^2+^  > Mg^2+^  > NH_4_^+^  > K^+^  > Na^+^ and Ca^2+^  > Mg^2+^  > Na^+^  > K^+^ ≈ NH_4_^+^ in diesel-high and gasoline-high exhausts, respectively. Although the relative abundance of Ca^2+^ increased as vehicle speed increased, the Ca^2+^ levels under idle (diesel: 2.49 μg/mg PM_2.5_; gasoline: 16.0 μg/mg PM_2.5_) and high speeds (diesel: 2.62 μg/mg PM_2.5_; gasoline: 18.4 μg/mg PM_2.5_) were similar (difference ≤ 15%). The relative anionic abundance was in the order of NO_2_^−^ > NO_3_^-^ ≈ PO_4_^3−^ > SO_4_^2−^ > Cl^−^ and NO_2_^−^ > PO_4_^3−^ > SO_4_^2−^ > NO_3_^-^ > Cl^−^ in diesel-high and gasoline-high exhausts, respectively.

Quantitation of PAHs in the dichloromethane extract of PM_2.5_ indicated that phenanthrene (Fig. 1) was the most abundant PAH in diesel-idle and gasoline-idle exhausts (Fig. 3B). Under idling operation, the order of the relative contents of PAHs was LMW-PAHs (45–73%) > MMW-PAHs (21–41%) > HMW-PAHs (7–14%) in the PM_2.5_ emitted from both diesel and gasoline engines. At high speed, the emissions contained mainly HMW-PAHs (> 54%). Among the HMW-PAHs, B[a]P, B[ghi]P, and I[cd]P were the top three constituents in the diesel-high exhaust. According to the total amount of PAHs in the particulates, the amounts of B[a]P, B[ghi]P, and I[cd]P in the diesel-high exhaust were greater than those in the gasoline-high exhaust. Thus, phenanthrene, B[a]P, B[ghi]P, and I[cd]P were further studied to reveal their contribution to the differential effects of exhausts on the expression of biomarkers IL-6, CYP1A1, HO-1, and NQO-1.

### Effects of the aqueous and dichloromethane extracts of PM_2.5_ on cell viability

**Figure 4:**
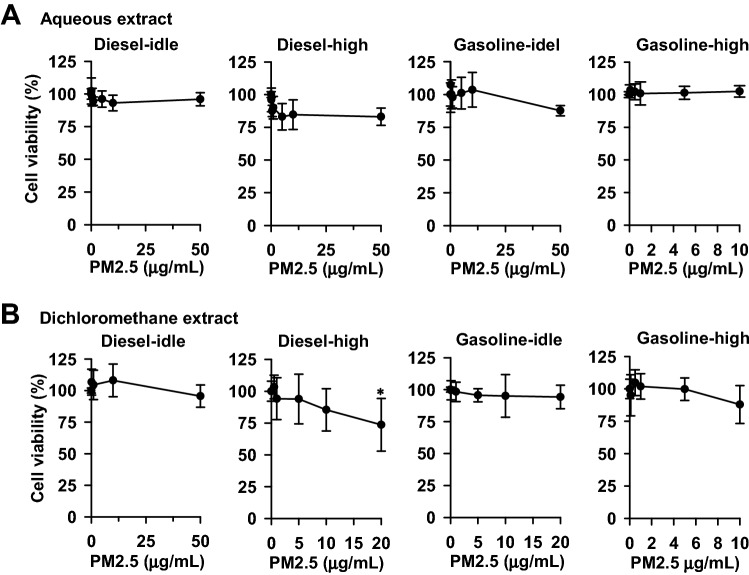
The influences of the aqueous (**A**) and dichloromethane (**B**) extracts of PM_2.5_ particulates on cell viability in A549 cells. After seeding, cells were cultured for 24 h and then exposed to the extracts of particulates for 48 h. Cell viability was monitored using MTT reduction assay as described in the Methods. Data represent the means ± SD of three separate experiments with triplicate determinations in each experiment. *p < 0.05 with a decrease > 20%.

For 48 h, A549 cells were exposed to the extracts at concentrations equivalent to PM_2.5_ exposure as indicated in Fig. 4. After exposure to the aqueous extracts, a significant decrease (> 20%) in cell viability was not observed (Fig. 4A). Among the dichloromethane extracts, exposure to 20 μg/mL of PM_2.5_, in the diesel-high exhaust caused a 24% decrease in cell viability (Fig. 4B), while the other exhausts did not cause a significant decrease. Thus, to prevent the influence of cell death, the cells were exposed to PM_2.5_ extracts at sub-toxic concentrations of aqueous and dichloromethane extracts at 10 μg PM_2.5_/mL, under which cell survival remained greater than 85%.

### Effects of fuel type and truck speed on biomarker induction by fuel emissions

**Figure 5:**
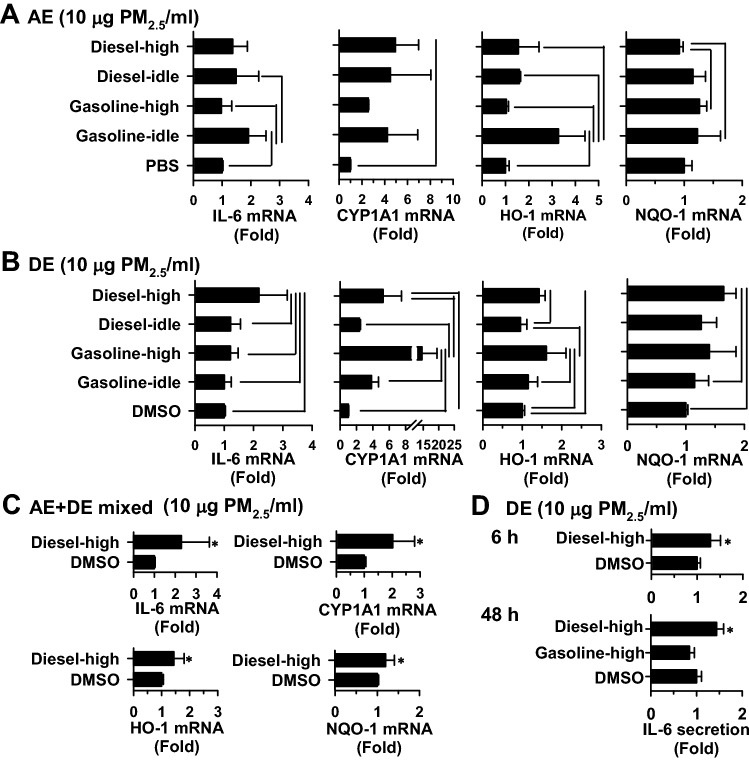
The biomarker transcript induction (**A**–**C**) and IL-6 secretion (**D**) by the extracts of PM_2.5_ particulates of fuel exhausts from engines under idling operation or high speed in A549 cells. In panels (**B**)–(**D**), cells were exposed to the aqueous extract (AE) and dichloromethane extract (DE) of PM_2.5_ particulates at a concentration as indicated for 48 h. After exposure, the mRNA levels of biomarkers were analyzed using real-time PCR analysis as described in the Methods. In panel (**E**), cells were exposed to the DE of diesel-high emission for 6 and 48 h as indicated. Data represent the means ± SD of three different experiments with duplicate determinations in each experiment. The lines between pairs of bars indicate that there are significant differences (*p* < 0.05) between two exposures in the one-way-ANOVA analysis with post-hoc Tukey’s test for multiple comparison. *p < 0.05 (compared to the control using Student’s *t*-test).

After 48-h exposure to the aqueous and dichloromethane extracts of PM_2.5_ (10 μg PM_2.5_/mL), compared to the vehicle control, the aqueous extract of gasoline-idle exhaust and the dichloromethane extract of diesel-high exhaust, significantly increased the IL-6 levels by 1.9 and 2.2 fold, respectively (Fig. 5A,B). Among the biomarkers, cellular CYP1A1 displayed the highest inducibility. The CYP1A1 transcript level significantly increased after exposure to the aqueous extract of diesel-high exhaust (5.0-fold, p < 0.05) (Fig. 5A), and the dichloromethane extracts of diesel-high (5.3-fold, p < 0.05) and gasoline-high exhausts (14.5-fold, p < 0.001) (Fig. 5B). Among the aqueous extracts, only the gasoline-idle exhaust increased HO-1 levels and none of the exhausts affected NQO-1 expression, compared to the vehicle control. The dichloromethane extracts of both gasoline-high and diesel-high exhausts caused significant increases in HO-1 expression, compared to vehicle control, and the induced HO-1 levels were significantly higher than those of idling operation. Among the dichloromethane extracts, only diesel-high exhaust significantly increased NQO-1 transcript levels. In addition, the cells were exposed to a mixture of aqueous and dichloromethane extracts of the diesel-high exhaust at 10 μg PM_2.5_/mL. The mixed extracts significantly induced the mRNA levels of IL-6, CYP1A1, HO-1, and NQO-1 (Fig. 5C). These results revealed that CYP1A1 is a very sensitive biomarker for fuel emissions, and the PAH-rich fraction of PM_2.5_ emitted from engines under high speed resulted in a greater increase in CYP1A1 and HO-1 than that under idle condition. The PAH-rich fraction from diesel-high exhaust, as well as a reconstituted mixture of the aqueous and dichloromethane extracts, stimulate biomarker expression in A549 cells.

### Effects of the fuel type on IL-6 secretion by the dichloromethane extracts of PM_2.5_ emitted under high speed

To further confirm the impact of increased IL-6 mRNA levels by the dichloromethane extract of PM_2.5_ at high speed, the secretion of IL-6 in the medium was determined. Consistent with the induction of IL-6 transcript (Fig. 5C), IL-6 secretion was significantly stimulated by 1.3- and 1.4-fold, after exposure to the extract of diesel-high exhaust for 6 h and 48 h, respectively (Fig. 5D). The stimulation of IL-6 secretion was not observed with the gasoline-high exhaust, which did not induce IL-6 expression in A549 lung cells (Fig. 5C).

### Protective effects of EGCG against the sub-toxic responses to the dichloromethane extract of diesel-high exhaust

**Figure 6:**
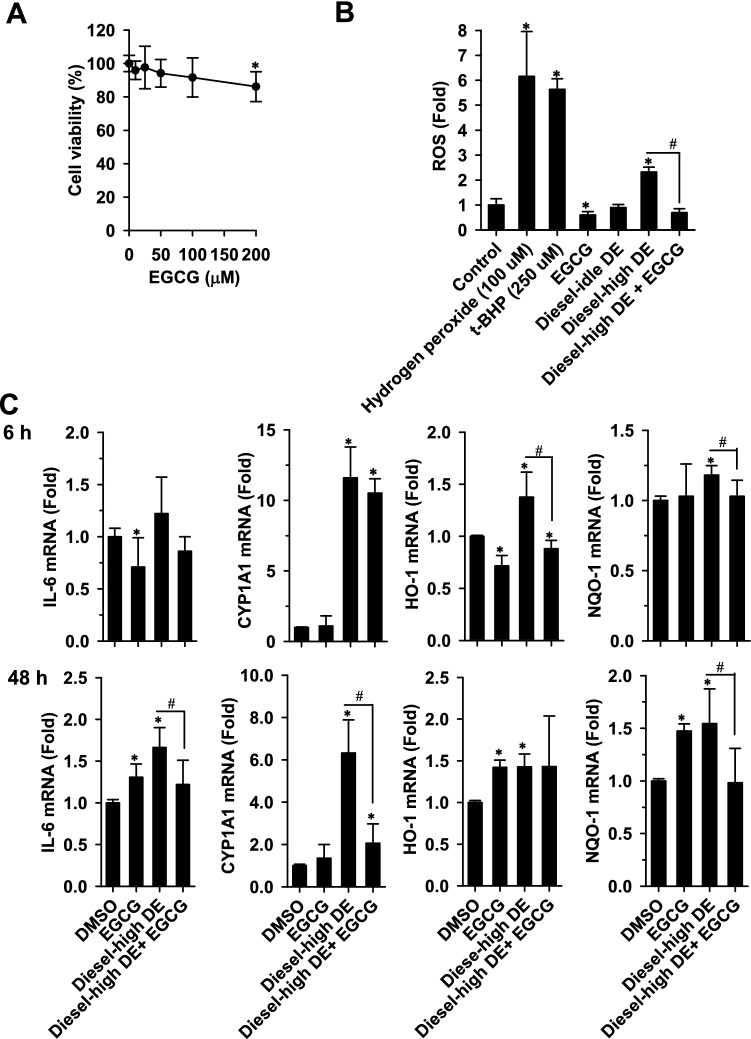
The protective effect of EGCG against the sub-toxic events induced by diesel-high exhaust in A549 cells. (**A**) Effects of EGCG on cell viability. Cells were exposed to increasing concentrations of EGCG for 48 h and cell survival was monitored by MTT assay. Data represent the means ± SD of three different experiments with triplicate determinations in each experiment. *p < 0.05 (**B**) EGCG reduced the cellular reactive oxygen species (ROS)-elevated by diesel exhaust exposure. Cells were exposed to 100 μM EGCG and 10 µg PM_2.5_/ml of the dichloromethane extracts (DEs) of diesel exhausts (alone or in combination). The generation of ROS was determined after 6-h exposure. Hydrogen peroxide and *t*-butyl hydroperoxide (*t*-BHP) were used as the positive exposure for ROS stimulation. (**C**) EGCG suppressed the potent biomarker-induction by DE of diesel-high exhaust. The mRNA levels of the exposure biomarkers were determined in cells exposed to the DE of the diesel-high exhaust with or without the co-exposure to EGCG for 6 and 48 h. In the determinations of ROS and mRNA levels, data represent the means ± SD of three different experiments with duplicate determinations. ^*^p < 0.05 compared to the DMSO exposure; ^#^*p* < 0.05 compared to the DE of diesel-high exhaust (Student’s *t*-test).

Effects of EGCG on cell growth was first studied to determine the exposure concentration of EGCG, at which cell growth was not affected in A549 cells. With 48-h of exposure to EGCG, cell viability decreased by 200 μM (86% survival) (Fig. 6A). Thus, cells were exposed to EGCG at a concentration ≤ 100 µM in the following studies. The 6-h exposure to hydrogen peroxide and t-BHP resulted in an increase (> five fold) in cellular ROS levels. In contrast, EGCG (100 μM) exposure decreased the basal cellular ROS levels by 40% (Fig. 6B). The 6-h exposure to the aqueous extracts of gasoline- and diesel-engine exhausts did not increase the generation of cellular ROS (data not shown). Similarly, exposure to the dichloromethane extract of the diesel-idle exhaust did not stimulate ROS generation. However, the dichloromethane extract of the diesel-high exhaust caused a twofold increase in ROS levels. This stimulation was diminished by the co-exposure to EGCG.

Due to the greater induction of IL-6 by the PAH-rich dichloromethane extract of the diesel-high exhaust, the effect of EGCG (100 µM) on the diesel-high exhaust-mediated induction of the biomarkers was further studied. EGCG exposure caused time-dependent bi-functional effects on IL-6 and HO-1 transcript levels and NQO-1 transcript increased after 48-h EGCG exposure (Fig. 6C). After 6-h of exposure, the extract of diesel-high exhaust stimulated CYP1A1, HO-1, and NQO-1 expression without affecting IL-6 expression (Fig. 6C). However, the diesel-high-induced increases in HO-1 and NQO-1 could be reduced by EGCG co-exposure, while EGCG could not affect the potent CYP1A1 induction diesel-high exhaust after 6-h short time exposure. By prolonging the exposure to 48 h, the diesel-high exhaust extract caused a 1.7-fold increase in IL-6 expression, and EGCG co-exposure significantly reduced IL-6 induction to a level comparable to the vehicle-control exposure. The elevation of transcript levels of CYP1A1 and NQO-1 by the extract also decreased after the cells were co-exposed to EGCG. Results showed the induction of the metabolic enzymes (CYP1A1, HO-1 and NQO-1) occurs shortly after the diesel-high exhaust exposure and the IL-6 stimulation required a longer time-period of exposure. Following the EGCG-decreased exhaust-mediated HO-1 and NQO-1 induction, EGCG ameliorated CYP1A1 and IL-6 induction by the exhaust extract.

### Effects of EGCG on the induction of IL-6, CYP1A1, HO-1 and NQO-1 by the main PAHs

**Figure 7:**
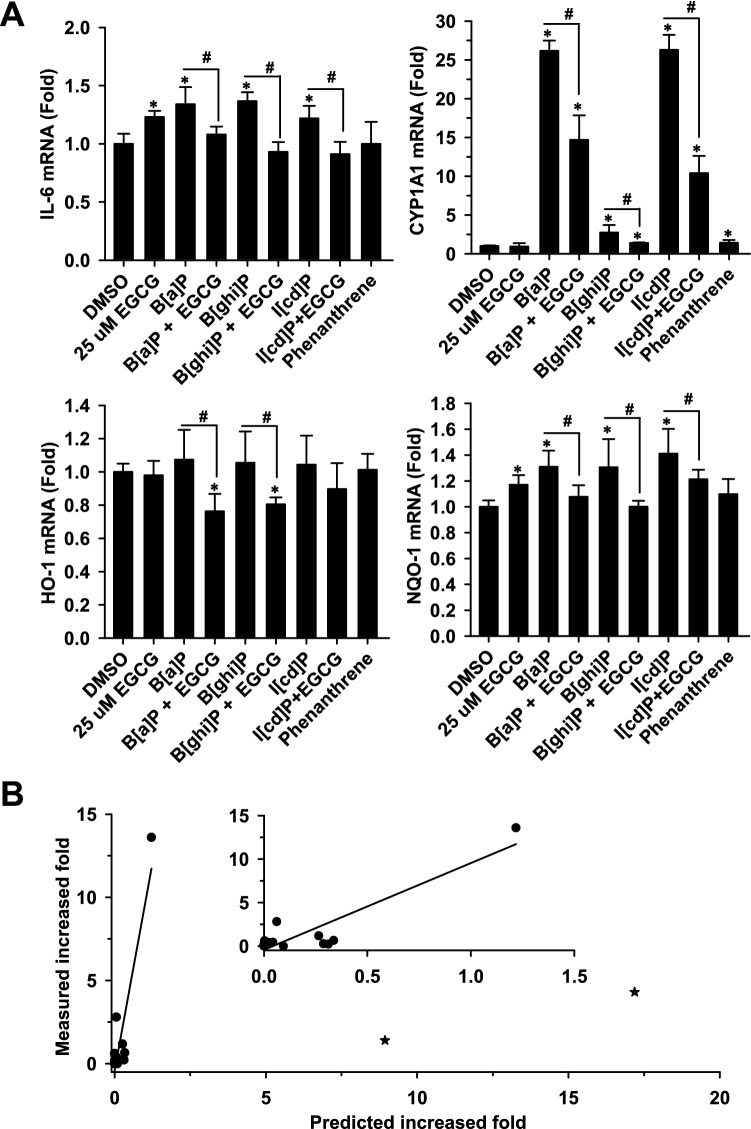
EGCG ameliorates the potent sub-toxic biomarkers induction by HMW-PAHs (**A**) and the significant correlation between predicted biomarker-induction through HMW-PAHs and the measured induction of biomarkers by the dichloromethane extracts (DEs) of truck exhausts (**B**). In panel (**A**), cells were exposed to PAHs (2 μM) alone or together with EGCG (25 μM) for 48 h. Data represent the means ± SD of three different experiments with duplicate determinations in each experiment. *, p < 0.05, compared to the control; ^#^*p* < 0.05, compared to the DE of diesel-high exhaust (Student’s *t*-test). (**B**) There was a positive correlation between predicted and measured (experimental) increases in the mRNA levels of IL-6, CYP1A1, HO-1 and NQO-1 by the gasoline/diesel emissions under idle and high speeds. The correlation between measured and predicted increases were analyzed using the GraphPad Prism software (version 3.02, GraphPad Software, San Diego, CA, USA) as described in the Methods. The solid line shows the best fit of a positive correlation following the equation: measured value = A + B × (predicted value) (r = 0.904). A and B were − 0.424 and 9.97, respectively. ★, outliers.

Due to the significant increases in the IL-6, HO-1 and NQO-1 transcript levels after 48-h exposure to 100 µM EGCG alone (Fig. 6C), EGCG exposure level was reduced to 25 µM to examine its effect on the biomarker induction by PAHs. With the decreased EGCG exposure from 100 to 25 µM, the increases in IL-6 and NQO-1 transcript levels reduced from 31 to 23% and 47% to 17%, respectively (Fig. 7A). CYP1A1 and HO-1 transcript levels remained unchanged after 25 µM EGCG exposure. To compare the biomarker-inductive effects of the main PAHs present in the dichloromethane extracts of the idle and high-speed exhausts, the effects of the LMW-PAH, phenanthrene, and the HMW-PAHs, B[a]P, B[ghi]P, and I[cd]P, on the cellular expression of IL-6, CYP1A1, HO-1, and NQO-1 were studied (Fig. 7A). According to the reported exposure concentration range of B[a]P for evoking biomarker induction and genotoxic potential^25–27^, the cells were exposed to these four PAHs at the same concentration of 2 μM for the purpose of comparison. The 48 h-exposure to 2 μM B[a]P, B[ghi]P and I[cd]P elevated the transcript levels of IL-6 by 1.2–1.4 fold. However, phenanthrene exposure did not affect the expression of IL-6. Phenanthrene, B[a]P, B[ghi]P and I[cd]P-exposure increased CYP1A1 transcript levels by 1.4-, 25.2-, 1.8- and 25.3-fold, respectively. In addition, B[a]P, B[ghi]P and I[cd]P-exposure increased NQO-1 transcript levels by 1.3-, 1.3- and 1.4-fold, respectively. The expression of HO-1 was not affected by the exposure to PAHs. The results showed that HMW-PAHs, B[a]P, B[ghi]P and I[cd]P caused more potent inductive effects on biomarkers than phenanthrene. However, the IL-6, CYP1A1 and NQO-1 induction by all HMW-PAHs was significantly reduced by the co-exposure to EGCG.

To examine the contribution of phenanthrene, B[a]P, B[ghi]P and I[cd]P to the differential effects of fuel emission under idling operation and high speed, relative increases in the biomarkers were predicted. The predicted and measured increases in transcript levels of IL-6, CYP1A1, HO-1, and NQO-1 (16 sets of values) showed a positive correlation with a Spearman’s correlation coefficient (r) of 0.691 (95% confidence interval: 0.283–0.888, p = 0.003). While the data were assumed to be sampled from Gaussian populations, the results of Pearson’s test showed no significant correlation with Pearson’s r of 0.238 (95% confidence interval: -0.292–0.657, p = 0.374), potentially due to two outliers. Following the removal of the two outliers, the predicted and measured increases in transcript levels of IL-6, CYP1A1, HO-1, and NQO-1 (14 sets of values) showed a positive correlation with a Spearman’s r of 0.582 (95% confidence interval: 0.057 – 0.855; p = 0.0289) and Pearson’s r of 0.904 (95% confidence interval: 0.719 – 0.970; p < 0.0001) (Fig. 7B). The results suggested that phenanthrene, B[a]P, B[ghi]P, and I[cd]P represent the principal PAHs for the differential responses of the biomarkers in lung cells after exposure to various exhausts.

## Discussion

In our previous study of PM_2.5_ in northern Taiwan, the results showed that traffic activity generates higher PM_2.5_ concentration (mg/m^3^) than long-range transport events and outdoor cooking (night market)^28^. The potential factors influencing the PM_2.5_ composition in vehicle emissions include fuel type, engine age, running distance, vehicle displacement (c.c.), wind speed, temperature, and humidity while collecting the exhaust^15,16,29,30^. In the gasoline exhausts of 15 cars (1328 ~ 2378 c.c.), the concentrations of PM_2.5_ in the emission were found to be 0.36–1.02 mg/m^3^^15^. In another study of the diesel exhausts from 15 heavy trucks (4009–11,149 c.c.), the PM_2.5_ levels in the emission were 3.88–16.50 mg/m^3^^16^. From those results, diesel emissions seemed to contain greater PM_2.5_ particulates than gasoline emissions. However, there was fourfold difference in the mean engine displacement of vehicles between these two reports. Our study is the first to demonstrate that a light diesel-truck (2835 c.c.) emitted a PM_2.5_ level of more than eightfold that of emission from a gasoline-truck (2000 c.c.). The emissions from one diesel truck at high speed (80 km/h) had a 94% increase in the particulate exhaust rate (mg/min) than that at low speeds (20 km/h)^31^. Our findings further indicated that engines under high speed emitted a PM_2.5_ level of 35%-108% higher than the engines under idling operation. Compared to speed, both our present findings, and that from previous studies, revealed that fuel type had a greater influence on the absolute amounts of PM_2.5_, emitted from vehicles.

Analysis of the ionic and metallic (Suppl. Fig. 1) composition of the emitted PM_2.5_, revealed that, among the truck exhausts, the PM_2.5_, in gasoline-idle exhaust, had the highest levels of total cation/anion and metals. When the gasoline and diesel engines changed from idling operation to high speed, the most abundant cations and anions changed from Na^+^ to Ca^2+^ and from NO_3_^-^ or PO_4_^3−^ to NO_2_^−^, respectively. The increased speed changed the most abundant metals in PM_2.5_, from sodium to aluminum (Suppl. Fig. 2). The relative abundance of several metals increased with the increased speed, e.g. abundance of zinc increased 23–26 fold. In this study, used trucks were studied and the same truck was used for the PM_2.5_ collections under idle and high-speed conditions. The components of PM_2.5_ could be from fuel combustion, crustal dust and other absorbed substances. The actual causes for the changes in the relative abundance of ions, such as Na^+^, and metals, such as Zn, in the PM_2.5_ remained unclear. Potential causes may include the differential engine-temperature and the relative contribution of crustal dust and fuel combustion to the PM_2.5_ emitted from the engines under different speed conditions.

In the PAH content of vehicle emissions, large variations exist based on different reports^30^. In previous studies on the determination of 21 PAHs, the mean concentrations of total PAHs (Σ_21_PAHs) were 206 ng/m^3^ and 5310 ng/m^3^ in the gasoline exhaust of cars (< 2378 c.c.) and diesel exhausts from heavy trucks (> 4009 c.c.), respectively^15,16^. Compared to the reported PAH contents in gasoline and diesel exhausts, our determinations of the total PAHs in PM_2.5_ emitted from light trucks were relatively low. Our findings revealed that the PM_2.5_ from diesel emissions contained greater total PAHs compared to gasoline emissions under idle or high-speed condition, and the diesel-idle exhaust generated the highest total PAH content in PM_2.5_ (135 ng/mg PM_2.5_). Our study is the first to show that under idling operation, LMW-PAHs was the most abundant type of PAH (37–45% of total PAHs), emitted from trucks with either diesel (with a catalyzed diesel particulate filter) or gasoline engine. Consistent with a previous report that indicated that the abundance of heavier PAHs increased while the speed of a heavy-duty diesel truck increased^18^, our findings further showed that HMW-PAHs became the most abundant type of PAH (54–87% of total PAHs) in the PM_2.5_ of not only diesel but also gasoline exhaust when the speed of light trucks increased to 70 km/h. Under idle condition, the temperature of gas flow would be lower than that under high-speed condition. Temperature of gas flow could affect the partition of LMW-PAHs between particle and gas phases, which may lead to the relatively lower phenanthrene abundance in high-speed exhausts^32^. Temperature of gas flow could be one of the factors for the differential PAH composition of exhausts under idle and high speeds. Vehicle speed appeared to be a determining factor for the ion/metal/PAH composition of the PM_2.5_ emitted from trucks.

The TEF values used for calculating B[a]P toxicity equivalents were estimated based on the tumor incidence or carcinogenicity of PAHs^33^. In the early stages of cancer development after PAH exposure, CYP1A1, HO-1, NQO-1, and IL-6 were identified as exposure biomarkers^34,35^. Among the aqueous extract of exhausts, gasoline-idle exhaust caused the highest increases in IL-6 and HO-1 expression. Compared to the biomarker induction caused by the PAH-rich dichloromethane extract of PM_2.5_ emissions, the aqueous extract-mediated increase was weak to moderate. Among the exhausts, PM_2.5_ in the diesel-idle exhaust had the highest levels of total PAHs (ng/mg PM_2.5_) and B[a]Peq (ng/mg PM_2.5_). However, under sub-toxic acute exposure to the exhaust extracts, the PAH-rich dichloromethane extract of diesel-high exhaust effected the highest increase in IL-6 transcript level. Exposure to diesel-high exhaust extract resulted in a greater increase in HO-1 compared to effect of exposure to the diesel-idle exhaust. CYP1A1 was significantly induced by the diesel-high exhaust extract, but not by the diesel-idle exhaust extract. The dichloromethane extract of gasoline-high exhaust also significantly induced CYP1A1 and HO-1, compared with gasoline-idle. The PAH-rich fraction of PM_2.5_, emitted from trucks under high speed, resulted in greater biomarker induction compared to trucks under idling operation. Compared to the most abundant LMW-PAHs in the diesel-idle exhaust, phenanthrene, the HMW-PAHs, B[a]P, B[ghi]P, and I[cd]P effected greater increases in IL-6, CYP1A1, and NQO-1 levels. Our estimates of the relative increases in biomarkers correlated well with the measured increases after the exposure of A549 cells to PAH-rich extracts. This indicates that the PAH-mediated biomarker induction by exhausts is primarily attributed to these HMW-PAHs, whose abundance increased when truck ran at high speed. The lack of HO-1 induction by the 3 main HMW-PAHs suggested the presence of other types of inducers in the dichloromethane extract of diesel-high exhaust. In addition to PAHs, nitro-PAHs and other organic compounds as the persistent organic pollutants (POPs), dibenzo-p-dioxins, dibenzofurans, and polychlorinated biphenyls (PCBs), are other classes of stimulators^27^.

It was demonstrated that AhR induction is linked to Nrf2 activated downstream targets, HO-1 and NQO-1^36^. Induction of CYP1A1 and NQO-1 is crucial for the degradation of harmful toxicants, including PAHs and quinones^37^. However, persistent AhR activation and subsequent oncogene activation can also lead to the increase of carcinogenic metabolite-induced DNA damage and the unbalanced promotion of cell proliferation^1,7,10^. EGCG exposure was reported to cause inconsistent regulation of Nrf2, HO-1, and NQO-1 expression in different cell types^38,39^. In consistence with that EGCG (100 μM EGCG, 24 h) increased the expression of HO-1 in A549 cells and A549 xenografts^40^, our findings revealed that EGCG decreased HO-1 expression after 6-h exposure and then induced HO-1 in a dose-dependent manner after 48-h exposure. The 6-h exposure to EGCG alone decreased the basal level of ROS and IL-6 expression. Co-exposure to EGCG reduced the ROS, HO-1 and NQO-1 level increased by PAH-rich dichloromethane extract of diesel-high-emitted PM_2.5_. Although both EGCG and the PAH-rich diesel-high exhaust extract induced the expression of IL-6 and NOQ-1 after 48-h exposure, co-exposure to EGCG reduced the stimulation by the diesel-high exhaust extract. The 6 and 48-h exposure to a high concentration of EGCG alone did not affect the basal CYP1A1 transcript level, but 48-h co-exposure to EGCG significantly decreased the diesel-high exhaust-mediated CYP1A1 induction due to its potential inhibitive effect on the AhR-signaling^19,20^. The time-dependent influence of EGCG on fuel-emission-induced biomarker responses was first reported in this study. EGCG decreased the diesel-high extract- and the HMW-PAH-stimulated IL-6, indicating the benefit of EGCG in ameliorating the IL-6 stimulation by diesel-high-emitted PM_2.5_.

In conclusion, our findings reveal that truck speed is a determining factor of the ionic, PAH and metallic composition of PM_2.5_. B[a]P, B[ghi]P and I[cd]P were identified to be the main PAHs facilitated by diesel-truck speed and caused potent induction of biomarkers in lung cells. The diesel-high exhaust-stimulated ROS and relevant HO-1 and NQO-1 levels were first attenuated by EGCG and then the elevated CYP1A1 and IL-6 levels decreased.

## Methods

### Chemicals and solvents

B[a]P, benzo[ghi]perylene (B[ghi]P), indeno[1,2,3-*cd*]pyrene (I[cd]P), and phenanthrene were purchased from Sigma-Aldrich (St. Louis, MO, USA). Deuterated PAHs (naphthalene-d_8_, 2-methylnaphthalene-d_10_, acenaphthylene-d_8_, phenanthrene-d_10_, anthracene-d_10_, fluoranthene-d_10_, benz[a]anthracene-d_12_, chrysene-d_12_, benzo[b]fluoranthene-d_12_, benzo[k]fluoranthene-d_12_, benzo[a]pyrene-d_12_, perylene-d_12_, Indeno[1,2,3-cd]pyrene-d_12_, dibenz[a,h]anthracene-d_14_, benzo[g,h,i]perylene-d_12_, dibenzo[a,i]pyrene-d_14_) and internal standard PAHs (acenaphthene-d_10_, pyrene-d_10_, benzo[e]pyrene-d_12_) were purchased from Wellington Laboratories Inc. (Guelph, Ontario, Canada). Dichloromethane, dimethyl sulfoxide (DMSO), and ethanol were purchased from Merck KGaA (Darmstadt, Germany). Nitric acid and hydrofluoric acid were purchased from J. T. Baker (Avantor, PA, USA).

### Sampling and sample preparation of mobile exhaust

The diesel emissions were collected from a used (over 5 years) light truck (diesel engine, stage III, 2002, engine displacement: 2835 c.c., Fuso Canter, with catalyzed diesel particulate filter DPX, Mitsubishi Motors, Tokyo, Japan) under both idling operation (diesel-idle) and 70 km/h (diesel-high). The standard sampling volume was 0.81–1.22 Nm^3^ with an average flow rate of 2.80–2.85 m/s. The gasoline emissions were collected from a gasoline engine (both idle (gasoline-idle) and 70 km/h (gasoline-high) in a truck (stage III, 1997, engine displacement: 2000 c.c., Freeca, Mitsubishi Motors, Tokyo, Japan). The standard sampling volume was 0.53–1.27 Nm^3^ and the average flow rate 1.12–6.96 m/s. In the open environment, a chassis dynamometer was used to control the speed of vehicles and the exhaust was collected for 2–4 h. The flue gas temperatures of diesel and gasoline engines under idle condition were around 42–51 °C. Under high-speed condition, a higher gas temperature (123–129 oC) of diesel and gasoline engines was observed. The emitted CO_2_ and O_2_ levels in the exhausts were 1.5–12.9% and 2.6–18.8%, respectively. The water content in gasoline engine exhaust (9.9–14.9%) was higher than that in diesel engine exhaust (1.6–5.4%). The filterable particulate matter (PM_2.5_) was collected on the Whatman quartz fiber filter (QM-A, 47 mm diameter; Merck KGaA, Darmstadt, Germany) using cyclonic separation (Apex Instruments Co Pvt. Ltd., West Bengal, India) with the precision of ± 3%. One sixteenth of the filter was cut using a pair of Nanocera scissors (Hola China, Shanghai, China), immersed in 10 mL deionized water and sonicated in a water bath at room temperature for 1 h. After centrifugation, the aqueous extract was then collected and lyophilized, while the filter was air-dried and placed in a Thimble filter (item no. 84, 31 mm (i.d.) × 35 mm × 120 mm, Advantec Toyo Kaisha, Ltd., Tokyo, Japan) for further preparation of dichloromethane extract. All apparatuses were then washed using acetone followed by dichloromethane. Soxhlet-extraction was performed using dichloromethane for 6 h without light. The extracts were concentrated using a rotor evaporator and dried under a stream of nitrogen gas at room temperature. The filter residues were used for further metallic determination (Suppl. Methods 1.1 and Results) and the detection rates were listed in Suppl. Table 1. A clean filter was extracted and analyzed as a blank sample for the chemical analyses. All extracts were stored at -20 °C in the dark.

### Analysis of chemicals

The aqueous extracts of PM_2.5_ samples were filtered using a glass syringe and a 20 mm polytetrafluoroethylene (PTFE) filter (Advantec Toyo Kaisha, Ltd., Tokyo, Japan). The filtrate was subjected to ion chromatography using Dionex ICS-1000 (Thermo Fisher Scientific Inc., Waltham, MA, USA) to determine the ionic content (recovery range of 88–104%)^28^ with a detection limit of 0.01–10.1 µg.

For the quantitation of PAHs, we analyzed the contents of 27 PAHs in the dichloromethane extracts of PM_2.5_ samples. Each PM_2.5_ extract was spiked with internal standards (deuterated PAHs, 50 μl of 50 pg/μl for each sample) before reconstitution with dichloromethane (40 mL). The reconstituted samples were sonicated for 15 min and then incubated at room temperature in the dark for 5 min; this was repeated three times. The sample solutions were filtered as previously described and then concentrated using a rotary evaporator in a thermostatic bath (Eyela, Tokyo, Japan) at 33 °C. The final solution volume was reduced to 1.0 mL under a nitrogen stream. The PAHs were separated and quantitated by gas chromatography-mass spectrometry (GC–MS/MS) using the TRACE 1300 GC & TSQ 8000 Evo Triple Quadrupole system (Thermo Fisher Scientific Inc.) equipped with a DM-5MS column (60 m × 0.25 mm × 0.25 μm, DiKMA Technol. Inc., Foothill Ranch, CA, USA) (Suppl. Table 2)^41,42^. A mixture of PAH standards (deuterated PAHs) was added to the blank extract and analyzed as previously described. Acenaphthene-d_10_, pyrene-d_10_, and benzo[e]pyrene-d_12_ were used as standards for the recovery determination. The recovery efficiency ranged from 72 to 95% and the limits of detection (LOD) ranged from 0.30 to 2.1 ng. To prevent underestimation, when the level of a PAH compound was lower than the LOD value, a LOD/2 value was assigned. The detection rates of ions and PAHs were shown in Suppl. Table 1. B[a]P equivalent (B[a]Peq) was calculated using Eq. 1, where i represents individual PAH compounds. The toxicity equivalent factor (TEF) of each PAH compound is listed in Suppl. Table 3.

1 $${\text{B}}[{\text{a}}]{\text{Peq}} = \Sigma [{\text{PAH}}_{i} ] \times {\text{TEF}}_{i}$$

### Cell culture, exposure, and viability

A549 cells were purchased from the Bioresource Collection and Research Center, Food Industry Research and Development Institute, Hsinchu, Taiwan. The characterization of the A549 cells was performed using short tandem repeat-polymerase chain reaction (PCR) using an AmpFLSTR Identifier PCR amplification kit (Applied Biosystems/Thermo Fisher, Foster City, CA, USA) and DNA profiling was conducted using an ABI 3730 Sequencer (Applied Biosystems Inc.). The cells (within 20 passages) were cultured in a F-12 K nutrient mixture (Kaighn’s Modification, Gibco, Thermo-Fisher Scientific Inc.) supplemented with 10% (v/v) fetal bovine serum (FBS) (Gibco, Thermo Fisher Scientific Inc) in a humidified atmosphere at 37 °C with 5% CO_2_. The cells were cultured overnight and then washed with phosphate-buffered saline (PBS) containing 150 mM NaCl, 2.7 mM KCl, 1.3 mM KH_2_PO_4_, and 8.1 mM Na_2_HPO_4_ (pH 7.4). They were then exposed to PAHs (in DMSO) or the PM_2.5_ extracts (aqueous extract in PBS and dichloromethane extract in DMSO) in the culture medium^43^. The control cells were exposed to the same concentration of PBS and DMSO (0.1%) as in the exhaust extracts/EGCG exposed cells. Cell viability was monitored by measuring the cellular reduction activity toward 3-(4,5-dimethyl-thiazol-2yl)-2,5-diphenyl tetrazolium (MTT)^44^. After overnight culture of 4000 cells/well in a 96-well plate, cells were exposed to exhaust extracts/EGCG for 48 h before cell viability determination. Cell viability was expressed as the relative reduction activity compared to the vehicle control.

### Real-time PCR analysis of the reverse-transcript of ribonucleic acid (RNA)

Total RNA of cells (6–48-h exhaust extract/EGCG exposure of the overnight culture of 3 × 10^5^ cells/well in a 6-well plate) was isolated using TRIzol reagent according to the manufacturer’s protocol (Invitrogen Co., CA, USA). RNA samples (2 μg) were subjected to reverse transcription (RT) at 42 °C for 1 h and then at 70 °C for 5 min, using genetically modified M-MuLV reverse transcriptase from the RevertAidTM First Strand cDNA Synthesis kit (Fermantas Inc., MD, USA). Quantitative real-time PCR analysis was then performed using primer sets (Suppl. Table 4) and SYBR Green (Roche Molecular Biochemicals, Mannheim, Germany). The amplification products were analyzed using a LightCycler 480 II/96 (Roche Diagnostics Ltd., Rotkreuz, Switzerland). The amplification conditions were as follows: 2-min pre-incubation at 95 °C followed by 45 cycles of 5 s at 95 °C, 10 s at 60 °C, and 15 s at 72 °C. The quantity of target cDNA in each sample was determined using the fractional PCR threshold cycle number (Ct value). Relative mRNA expression levels were normalized to glyceraldehyde 3-phosphate dehydrogenase (GAPDH) expression and the target cDNA was calculated using 2^−(Ct(target) –Ct(GAPDH))^.

### IL-6 secretion and ROS determination

For the determination of the release of IL-6, the medium was collected after exposure and centrifuged at 6000×*g* for 5 min at 4 °C. The supernatant was subjected to an ELISA using a commercially available ELISA kit (R&D Systems, Inc., Minneapolis, MN, USA). Cellular ROS levels were determined after 6-h pollutant exposure using the Cellular ROS Assay Kit (ab113851; *t*-butylhydroperoxide (t-BHP) included) purchased from Abcam plc (Cambridge, UK) and expressed as the relative level compared to the vehicle control. Hydrogen peroxide and t-BHP were used as positive controls for ROS generation.

### Correlation analysis

To examine the contributions of phenanthrene, B[a]P, B[ghi]P, and I[cd]P to differential biomarker-inductive effects of the dichloromethane extract of different PM_2.5_ emissions, the correlation between measured increases and the predicted relative increases in biomarkers were analyzed. The relative increase (RI) in a biomarker by a sample of PM_2.5_ was predicted using Eq. 2, in which the concentration of individual PAH was its molar concentration in the 10 μg/mL PM_2.5_. Spearman’s and Pearson’s correlation tests were performed to examine the relationship between measured and predicted increases, using the GraphPad Prism software (version 3.02, GraphPad Software, San Diego, CA, USA).

2 $${\text{RI}} = ({\text{Increase}}_{{{\text{phenanthrene}}}} \times [{\text{phenanthrene}}]) + ({\text{Increase}}_{{{\text{B}}[{\text{a}}]{\text{P}}}} \times [{\text{B}}[{\text{a}}]{\text{P}}]) + ({\text{Increase}}_{{{\text{B}}[{\text{ghi}}]{\text{P}}}} \times [{\text{B}}[{\text{ghi}}]{\text{P}}]) + ({\text{Increase}}_{{{\text{I}}[{\text{cd}}]{\text{P}}}} \times [{\text{I}}[{\text{cd}}]{\text{P}}]).$$

### Statistical analyses

Differences between data sets (> 2) were analyzed by one-way analysis of variance (ANOVA) followed by a post-hoc Tukey’s test for multiple comparisons (SPSS statistics, version17.0, SPSS Inc., USA). The differences between two exposures were analyzed using the Student’s *t*-test. Statistical significance was set at *P* < 0.05.

## Supplementary Information

Supplementary Information.

## Data Availability

The datasets used and/or analysed during the current study available from the corresponding author on reasonable request.

## Abbreviations

AhR: Arylhydrocarbon receptor
B[a]P: Benzo[*a*]pyrene
B[ghi]P: Benzo[*ghi*]perylene
*t*-BHP: *t*-Butylhydroperoxide
CYP: Cytochrome P450
DMSO: Dimethyl sulfoxide
LMW: Low-molecular weight
HMW: High-molecular weight
HO-1: Hemeoxygenase-1
I[cd]P: Indeno[1,2,3-*cd*]pyrene
IL-6: Interleukin-6
MMW: Medium-molecular weight
NQO-1: NAD(P)H-quinone oxidoreductase-1
Nrf2: Nuclear factor-erythroid 2-related factor 2
PAH: Polycyclic aromatic hydrocarbon
PM: Particulate matter
