# Supplementary material for: Sub-toxic events induced by truck speed-facilitated PM2.5 and its counteraction by epigallocatechin-3-gallate in A549 human lung cells
Source: Sci Rep. 2022 Sep 2;12:15004. doi: 10.1038/s41598-022-18918-x (PMC9440210; doi:10.1038/s41598-022-18918-x)
Supplement: Supplementary file 1 — Supplementary Information. [file 41598_2022_18918_MOESM1_ESM.docx]

**Supplementary materials**

1. **Supplementary Methods**:

*1.1 Preparation of the metallic extract and analysis of the metallic content of the PM_2.5_ samples*:

After dichloromethane extraction, the filter residues were digested with a mixture of 60% nitric acid/48% hydrofluoric acid (2:1, 6 mL) and a microwave digestion system (MARS Xpress, CEM Corporation, Matthews, NC, USA). Neutralization using a 3 M sodium hydroxide solution afforded the metallic extract. A clean filter was extracted and analyzed as a blank sample for the chemical analyses. All extracts were stored at -20^o^C in the dark. Inductively coupled plasma mass spectrometry (ICP-MS) (Elan 6100, PerkinElmer Instruments, Akron, OH, USA) was used for the analysis of metals (Hsu et al., 2021). Element standards were prepared from a stock standard (SPEX CertiPrep, Metuchen, NJ, USA) in 5% HNO_3_. The recovery was ranged from 90% to110% and the detection limit was 2.3-3.4 ng. The blank value of each selected element on the blank filter was subtracted from the analyzed values from the real samples.

Supplementary Table 1. The detection rates of ions, PAHs and metals in 4 exhausts.

| Ion | Detection rate (%) | PAH | Rings | Detection rate (%) | Metal | Detection rate (%) |
| --- | --- | --- | --- | --- | --- | --- |
| Na^+^ | 100 | Naphthalene | 2 | 50 | Al | 100 |
| K^+^ | 25 | 2-Methylnaphthalene | 2 | 75 | Fe | 100 |
| Ca^2+^ | 100 | Acenaphthylene | 3 | 100 | Na | 100 |
| Mg^2+^ | 100 | Acenaphthene | 3 | 100 | Mg | 100 |
| NH_4_^+^ | 75 | Fluorene | 3 | 100 | K | 0 |
| Cl^-^ | 100 | Anthracene | 3 | 100 | Ca | 100 |
| NO^2-^ | 100 | Phenanthrene | 3 | 100 | Sr | 100 |
| NO^3-^ | 100 | Fluoranthene | 4 | 100 | Ba | 100 |
| PO_4_^3-^ | 100 | Pyrene | 4 | 100 | Ti | 100 |
| SO_4_^2-^ | 100 | Benzo[*c*]fluorene | 4 | 100 | Mn | 100 |
|  |  | Benz[*a*]anthracene | 4 | 100 | Co | 100 |
|  |  | Chrysene | 4 | 100 | Ni | 100 |
|  |  | 5-Methylchrysene | 4 | 25 | Cu | 100 |
|  |  | Cyclopenta[*c,d*]pyrene | 5 | 100 | Zn | 100 |
|  |  | Benzo[*b*]fluoranthene | 5 | 100 | Mo | 100 |
|  |  | Benzo[*j*]fluoranthene | 5 | 50 | Cd | 100 |
|  |  | Benzo[*k*]fluoranthene | 5 | 50 | Sn | 100 |
|  |  | Benzo[*e*]pyrene | 5 | 50 | Sb | 100 |
|  |  | Benzo[*a*]pyrene | 5 | 50 | Tl | 0 |
|  |  | Perylene | 6 | 25 | Pb | 0 |
|  |  | Dibenz[*a,h*]anthracene | 6 | 25 | V | 75 |
|  |  | Indeno[1,2,3-*cd*]pyrene | 6 | 100 | Cr | 100 |
|  |  | Benzo[*ghi*]perylene | 6 | 75 | As | 50 |
|  |  | Dibenzo[*a,l*]pyrene | 6 | 25 | Y | 100 |
|  |  | Dibenzo[*a,e*]pyrene | 6 | 25 | Se | 50 |
|  |  | Dibenzo[*a,i*]pyrene | 6 | 0 | Zr | 100 |
|  |  | Dibenzo[*a,h*]pyrene | 6 | 0 | Ge | 100 |
|  |  |  |  |  | Rb | 100 |
|  |  |  |  |  | Cs | 50 |
|  |  |  |  |  | Ga | 100 |
|  |  |  |  |  | La | 100 |
|  |  |  |  |  | Ce | 100 |
|  |  |  |  |  | Pr | 100 |
|  |  |  |  |  | Nd | 100 |
|  |  |  |  |  | Sm | 100 |
|  |  |  |  |  | Eu | 100 |
|  |  |  |  |  | Gd | 100 |
|  |  |  |  |  | Tb | 100 |
|  |  |  |  |  | Dy | 100 |
|  |  |  |  |  | Ho | 100 |
|  |  |  |  |  | Er | 100 |
|  |  |  |  |  | Tm | 100 |
|  |  |  |  |  | Yb | 100 |
|  |  |  |  |  | Lu | 100 |
|  |  |  |  |  | Hf | 100 |
|  |  |  |  |  | U | 100 |

The detection rate was the percentage of the number of samples with detected values in total 4 exhaust samples.

Supplementary Table 2. The method of GC-MS/MS in the analysis of PAHs.

| Mode | Splitless, purge flow rate: 30 mL/min; purge time: 2.05 min |
| --- | --- |
| Injection volume | 1.5 μL |
| Carrier gas | Helium |
| Carrier gas flow rate | 1.7 mL/min |
| Column | DB-5 MS (60 m 🞨 0.25 mm🞨 0.25μm, DiKMA Technol. Inc., Foothill Ranch, CA, USA) |
| Initial oven temperature | 70℃ |
| Heating conditions | Initial temperature at 70 ℃ for 5 min and then increased to 200 ℃ (stay for 18.9 min), followed by 280 ℃ for 1.6 min, 320 ℃ for 5 min, and 320 ℃ for 12.5 min |
| Final oven temperature | 320 ℃ |
| Detection | Electron ionization mode  MS transfer line temp.: 300  Ion source temp.: 320  Collision Energy: 38 eV |
| Quantitation | Selected Reaction Monitoring (SRM) |

Supplementary Table 3. The toxic equivalency factor (TEF) or relative potency factor (RPF) values of polycyclic aromatic hydrocarbons (PAHs) used in the calculation of benzo(a)pyrene equivalent

| PAH | Rings | TEF/RPF value | Reference |
| --- | --- | --- | --- |
| Acenaphthene | 3 | 0.001 | Nisbet and LaGoy, 1992 |
| Acenaphthylene | 3 | 0.001 |  |
| Anthracene | 3 | 0.01 |  |
| Benz[a]anthracene | 4 | 0.1 |  |
| Benzo[b]fluoranthene | 5 | 0.1 |  |
| Benzo[k]fluoranthene | 5 | 0.3 |  |
| Benzo[a]pyrene | 5 | 1 |  |
| Benzo[ghi]perylene | 6 | 0.01 |  |
| Chrysene | 4 | 0.01 |  |
| Dibenz[a,h]anthracene | 5 | 1 |  |
| Fluoranthene | 4 | 0.001 |  |
| Fluorene | 3 | 0.001 |  |
| Indeno[1,2,3-cd]pyrene | 6 | 0.1 |  |
| Naphthalene | 2 | 0.001 |  |
| Phenanthrene | 3 | 0.001 |  |
| Pyrene | 4 | 0.001 |  |
| Benzo[c]fluorene | 4 | 20 | Minnesota Department of Health (MDH), 2016  Morisaki et al., 2016 |
| Benzo[j]fluoranthene | 5 | 0.3 | Minnesota Department of Health (MDH), 2016 |
| Dibenzo[a,e]pyrene | 6 | 0.4 |  |
| Dibenzo[a,h]pyrene | 6 | 0.9 |  |
| Dibenzo[a,i]pyrene | 6 | 0.6 |  |
| Dibenzo[a,l]pyrene | 6 | 30 |  |
| 5-Methylchrysene | 4 | 1 |  |
| Cyclopenta[c,d]pyrene | 5 | 0.1 | Varlet et al., 2007 |
| Benzo[e]pyrene | 5 | 0.01 | Samburova et al., 2017 |
| 2-Methylnaphthalene | 2 | 0.001 |  |
| Perylene | 5 | 0.001 |  |

Supplementary Table 4. The primer sets used in the real-time PCR analyses.

| Genes | NCBI Reference sequence | Forward | Reverse | Base pair |
| --- | --- | --- | --- | --- |
| IL-6 | NM_000600.5 | ccagagctgtgcagatgagt | agttgtcatgtcctg | 165 |
| HO-1 | NM_002133.3 | aagactgcgttcctgctcaac | aaagccctacagcaactgtcg | 195 |
| NQO-1 | NM_000903.3 | ggcagaagagcactgatcgta | tgatgggattgaagttcatggc | 145 |
| CYP1A1 | NM_000499.5 | gtcatctgtgccatttgctttg | caaccacctccccgaaattatt | 97 |
| GAPDH | NM_002046.7 | cggagtcaacggatttggtcgtat | agccttctccatggtggtgaagac | 307 |

1. **Supplementary Results**

2.1 *The contents of metals in PM_2.5_ of the fuel emissions*:

Regarding PM_2.5_ composition, gasoline-idle exhaust contained the highest levels (μg/mg PM_2.5_) of metals. Determination of the metals revealed that the total metal content were in the order of gasoline-idle > gasoline-high ≅ diesel-idle > diesel-high exhaust (Suppl. Fig. 1). The contents of K, Tl and Pb were below the detection limit in all vehicle exhausts. Analysis of the metallic extract showed that aluminum, calcium, iron, magnesium, and sodium were the top 5 abundant metals in all fuel emissions (Suppl. Fig.2). Among them, the relative abundance of calcium (18-24%) and magnesium (7-10%) had the least changes. In both the gasoline and diesel exhausts, sodium (54-59%) and aluminum (44-48%) were the most abundant metals in the exhaust of trucks under idle and high speeds, respectively. When the truck speed changed from idling operation to high speed, the relative abundance of Ba (from < 0.1% to 0.6-0.8%), Mo (from 0.2% to 2%), Ti (from 0.1-0.3% to 0.6%), Zn (from < 0.3% to 1-8%), and Zr (from < 0.1% to 1%) increased in both the diesel and gasoline emissions. The relative abundance of metals was apparently affected by truck speed.

Supplementary Figure 1. The total metallic contents in the PM_2.5_ emitted from truck under idling operation and high speed.

Supplementary Figure 2. The total contents and composition of metals in PM_2.5_ particulates collected from trucks with gasoline or diesel engine under idle and high speeds.

**References**:

Hsu, C.Y., Chi, K.H., Wu, C.D., Lin, S.L., Hsu, W.C., Tseng, C.C., Chen, M.J., Chen, Y.C., 2021. Integrated analysis of source-specific risks for PM_2.5_-bound metals in urban, suburban, rural, and industrial areas. Environ. Pollut. 275, 116652. https://doi.org/10.1016/j.envpol.2021.116652

Minnesota Department of Health, 2016. Guidance for evaluating the cancer potency of polycyclic aromatic hydrocarbon (PAH) mixtures in environmental samples. Minnesota Department of Health, Saint Paul, M.N., USA https://www.health.state.mn.us/communities/environment/risk/docs/guidance/pahguidance.pdf.

Morisaki, H., Nakamura, S., Tang, N., Toriba, A., Hayakawa, K., 2016. Benzo[c]fluorene in urban air: HPLC determination and mutagenic contribution relative to benzo[a]pyrene. Anal. Sci., 32, 233-236. https://doi.org/10.2116/analsci.32.233.

Nisbet, I.C.T., LaGoy, P.K., 1992. Toxic equivalency factors (TEFs) for polycyclic aromatic hydrocarbons (PAHs). Regul. Toxicol. Pharmacol., 16, 290-300.

Samburova, V., Zielinska, B., Khlystov, A., 2017. Do 16 polycyclic aromatic hydrocarbons represent PAH air toxicity? Toxics, 5, 17. https://doi.org/10.3390/toxics5030017.

Varlet, V., Serot, T., Monteau, F., Bizec B.L., Prost, C., 2007. Determination of PAH profiles by GC–MS/MS in salmon processed by four cold-smoking techniques. Food Addit. Contam. 24, 744-757. https://doi.org/10.1080/02652030601139946.
